# Unhealthy weight among young children in the Middle East and North African region

**DOI:** 10.1017/S1368980023001684

**Published:** 2023-11

**Authors:** Rebecca Jones-Antwi, Solveig A Cunningham

**Affiliations:** Hubert Department of Global Health, Emory University, Atlanta, GA 30322, USA

**Keywords:** Middle East and North Africa, Wasting, Overweight, Child nutritional status

## Abstract

**Objective:**

To understand early-life growth in the Middle East and North Africa (MENA) region, and how it has changed over time, we estimated the prevalence of wasting and overweight at ages under 5 years.

**Design:**

Cross-sectional data from twenty-nine Demographic and Health Surveys with direct anthropometric data and parent-reported demographic information were examined. The study utilised the WHO Child Growth Standards to classify overweight (weight-for-height z-score ≥ 2 sd above the median), wasting (weight-for-height z-score ≤ 2 sd below the median) and unhealthy weight defined as either wasting or overweight.

**Setting:**

Nationally representative for nine of the MENA countries (Armenia, Azerbaijan, Egypt, Jordan, Mauritania, Morocco, Tunisia, Turkey and Yemen).

**Participants:**

Children under age 5 from nine MENA countries between 1987 and 2016 (*n* 155 961).

**Results:**

Across the region, at the most recent time point, between 7·3 and 23·6 % of children experienced unhealthy weight (Jordan – 7·3 %, Egypt –23·6 %); 1·7 and 16·6 % had wasting (Turkey, Yemen) and 2·0 and 15·0 % had overweight (Yemen, Egypt). Overweight was more common than wasting in all countries except Yemen and Mauritania. Between 1987 and 2016, the prevalence of unhealthy weight in the region increased (10·0–18·4 %) due to increases in both wasting and overweight. Boys had a higher prevalence of unhealthy weight than girls.

**Conclusion:**

Undernutrition continues to be a problem in some countries in the MENA region, and overnutrition is emerging as a health concern in many countries in the region. Countries in the region must advance programmes that reduce undernutrition while not overlooking or inadvertently promoting overnutrition.

Undernutrition and overnutrition in childhood are both associated with adverse health in childhood and later in life^(1)^. The 2013 Lancet Maternal and Child Nutrition Series noted the emergence of overnutrition in low- and middle-income countries, while also documenting the unfinished agenda in undernutrition^(1)^. Growth patterns during the first years of life are important for long-term health, including the onset of chronic diseases later in life^(2)^. For example, a systematic review of growth in infancy and subsequent health concluded that high BMI during the first 5 years of life is associated with adult obesity and early onset of metabolic syndrome^(3)^. In developing countries, 52·5 % of all deaths of young children (0–5 years) were attributable to malnutrition, including both over- and undernutrition^(4,5)^. Major improvements have been made over the past 20 years in reducing undernutrition among children under age 5^(6,7)^; yet, in 2015, 156 million children under 5 were chronically undernourished^(8)^. At the same time, the global prevalence of overweight among children under 5 has increased modestly with heterogeneous trends in low- and middle-income regions^(9)^. The Middle East and North Africa (MENA) region has been identified as a region with high prevalence of overweight and obesity among adults^(10)^. Indeed, more than half of adult women in Libya, Qatar and Kuwait had obesity in 2014.

Children in the MENA region may be affected both by under- and overnutrition. Child wasting, defined as low weight-for-height, was the leading cause of disability-adjusted life years in Afghanistan, Somalia and Yemen, based on data as current as 2013^(11)^. In Morocco in 2002, between 25 and 38 % of children up to age 5 years were estimated to be underweight (defined as BMI-for-age z-score more than two sd below the median)^(12)^. In Egypt in 2014, 8 % of children up to age 5 years were estimated to be wasted; wasting was associated with child characteristics (birth weight, gender), mother’s characteristics (age, education), father’s education and contextual factors (household wealth; urbanicity)^(13)^. At the same time, in 2014, 17 % of children under age 5 were at-risk-of-overweight or overweight^(14)^. In Turkey, at-risk-of-overweight and overweight increased from 1993 to 2013 for this age group^(15)^. In Iran, however, only 5% of children aged 12–18 years were overweight and underweight, respectively, in 2011–2012^(16)^. These data suggest that, from a regional perspective, both under- and overnutrition should be kept in focus for research and programmes.

The countries of the MENA represent a complex socio-geographic region, linked by broad similarities of climate, geography, historical context and cultural values^(17)^. The region consists of high-, middle- and low-income countries. Different definitions of the region have been proposed. Some include the twenty-one members of the Arab League (Algeria, Bahrain, Comoros, Djibouti, Egypt, Iraq, Jordan, Kuwait, Lebanon, Libya, Mauritania, Morocco, Oman, Palestine, Qatar, Saudi Arabia, Somalia, Sudan, Syria, Tunisia, United Arab Emirates and Yemen); some additionally include Israel, Iran and Turkey; broader definitions also include Armenia, Azerbaijan, Cyprus, Georgia, Mauritania and Somalia^(18–20)^. Notable defining characteristics among MENA countries include combinations of some of the following: largely Muslim populations; Arab-speaking; being previously part of the Ottoman Empire and later divided by the British and French empires and availability of oil resources^(21–23)^. Some countries in the region have experienced protracted armed conflicts and political unrest, with resulting humanitarian crises and out-migration since World War II^(24)^.

Increases in non-communicable disease mortality between 1991 and 2018 have been documented in some of the countries in the region (Lebanon, Bahrain, Egypt and Tunisia) and may well be occurring in others. These increases have generally been attributed to smoking, diet, physical inactivity and obesity^(25)^. In many countries in the region, public health policy responses to increases in chronic disease prevalence have been slow. Some policies in the region appear discordant with social, economic and political circumstances, for example, food fortification of rice flour in Egypt^(25,26)^. More recent policies in the area have focused on both sides of the malnutrition burden^(26,27)^.

This study uses all the nationally representative Demographic and Health Survey (DHS) data from the MENA region over time to provide national and regional estimates of unhealthy weight among young children. DHS data are well suited for comparisons across countries and over time because they use standardised survey instruments and sample designs. We calculate, for children under age 5 years across the region and in each country, current levels of overweight, wasting and overall unhealthy weight and differences in each of these between boys and girls.

## Methods

### Participants and settings

Data are from the DHS, nationally representative cross-sectional surveys fielded in low- and middle-income countries since 1985 to collect information on the health of women and young children, including direct anthropometric measurements^(28)^. Using the broadest definition of the MENA region from the definitions listed above, DHS data were available for nine of the countries, with twenty-nine surveys administered between 1987 and 2016: Armenia (2000, 2005, 2010, 2015–2016), Azerbaijan (2006), Egypt (1988, 1992, 1995, 2000, 2003, 2005, 2008, 2014), Jordan (1990, 1997, 2002, 2009, 2012), Mauritania (2000-2001), Morocco (1987, 1992, 2004), Tunisia (1990), Turkey (1993, 1998, 2003, 2013) and Yemen (1992, 2013). Data from Afghanistan (2015), Jordan (2007 and 2018) Syria (1989–1990) and Turkey (2008) could not be included because anthropometric data on children were either not collected or deemed unreliable by DHS and not released; data from Turkey (2018) have not yet been released^(28,29)^.

DHS sample women aged 15–49 years and collect data about them and their children under 5 years living in the same house. DHS utilise a three-stage sampling design with random selection at each stage: the first stage involves selecting subnational clusters, typically drawn from census files^(30)^, the second stage selecting households within the selected clusters the third stage selecting women within the selected households.

This analysis is representative of children under 5 years old in most of the included countries and children under 4 years old in Tunisia in 1990 and Egypt in 1988. Analyses involved two analytic samples. First, we estimated the current burden of under- and overnutrition using the most recent survey in each of the nine countries with available data, resulting in a sample of 56 477 children. Second, we evaluated trends in under- and overnutrition over time in all nine countries, with a sample of 155 961 children.

### Measurements and variables

DHS anthropometric data collection and procedures are standardised and have been described elsewhere^(31)^. We evaluated children’s anthropometric measures using the WHO Child Growth Standard^(32,33)^. We created variables based on these standards for weight-for-height z-score for sex and age. Overweight is set to 1 when the weight-for-height z-score is equal to or more than two sd above the median of the WHO standard^(32)^. Wasting is set to 1 when the weight-for-height z-score is equal to or less than two sd below the median of the WHO standard. Normal weight is set to 1 when the weight-for-height z-score is less than two sd below or less than one sd above the median of the WHO standard. DHS data are pre-cleaned for outliers and biologically implausible values of height and weight; these are flagged and were dropped from analyses^(34)^.

We define unhealthy weight as either wasting or overweight to capture the prevalence of malnutrition across the weight spectrum. Wasting is the most sensitive measure of acute malnutrition, but, in additional analyses, we also evaluated underweight (BMI-for-age z-score ≤ 2 sd below the median) and stunting (height-for-age z-score < 2 sd below the median).

### Analysis

Using STATA version 13.0 software (STATA Corp), we conducted analyses for each country and for the region. For country-specific analyses, we used standard DHS survey adjustments to generate nationally representative estimates. To create weights for the entire region, the files for each country were de-normalised, and the sample weight was adjusted for overall population (i.e. all countries pooled together) using the Census Bureau International Programs database^(35)^.

For each country and for the entire region, means were calculated for each variable. To evaluate trends over time in prevalence for the region, we calculated the regional prevalence every 3 years where individuals from any country with data within that 3-year range were included as part of the denominator. Chi-square tests were done to evaluate differences between age, sex and weight categories across the region and within countries. A χ^2^ Cochran–Armitage linear trend test was used to test for time trends in weight^(36)^. Trend analyses were done at the regional level to evaluate changes over time in overweight, underweight, stunting and unhealthy weight for children under age 5. Significance was set at *P* < 0·05.

To gain a better understanding of social patterns of unhealthy weight, we stratified the prevalence of wasting and overweight within each country’s most recent data by mother’s education, a measure of social status and resources, as follows: basic/no education; primary; secondary and higher. Armenia combined primary and secondary into one category and we kept it this way. This variable was not available for Yemen, which was consequently excluded from this analysis.

## Results

The survey-adjusted weight status of children under 5 years, by age (0–1, 1–2 and 2–5 years), at the most recent data wave from each country is shown in Table 1. The proportion of children under age 5 years who were classified as wasted ranged between 16·6 % in Yemen (2013) and 1·7 % in Turkey (2013). The proportion of children under age 5 years who were overweight in MENA countries with available data ranged between 15·0 % in Egypt (2014) and 2·0 % in Yemen (2013). Generally, the prevalence of wasting decreased with age; for example, in Yemen, 22·9 % of children aged zero to one year were wasted, but this number fell to 13·1 % for children at 2–5 years. Younger children (0–1 years) had higher wasting prevalence across most countries. Overweight prevalence did not have a clear age pattern.

**Table 1:** Distribution across weight categories among children under age 5 years in the Middle East and North African region at the most recent year of national data collection (%)

	Normal weight		Overweight		Wasting	
Armenia, 2015						
0–1 years	55·2	48·48, 61·70	11·4	8·33, 15·37	4·4	2·75, 7·04
1–2 years	49·3	42·44, 56·29	17·0	12·56, 22·55	0·6	0·11, 2·86
2–5 years	61·4	57·14, 65·43	12·8	10·29, 15·71	5·1	3·66, 7·08
Total	57·7	54·29, 61·07	13·3	11·17, 15·85	4·1	3·02, 5·41
Azerbaijan, 2006						
0–1 years	62·3	57·53, 66·93	11·0	9·31, 14·43	13·4	10·46, 17·12
1–2 years	65·1	61·66, 68·36	9·2	7·40, 11·49	4·6	3·35, 6·34
2–5 years	64·3	60·83, 67·67	15·5	13·10, 18·29	3·7	2·57, 5·33
Total	64·2	62·05, 66·32	12·0	10·67, 13·57	6·1	5·14, 7·29
Egypt, 2014						
0–1 years	55·4	53·03, 57·73	16·4	14·70, 18·26	10·9	9·2, 12·6
1–2 years	53·8	52·11, 55·46	15·1	13·83, 16·47	8·8	7·81, 9·98
2–5 years	58·0	56·00, 59·97	14·3	12·76, 15·90	6·9	5·99, 7·90
Total	55·7	54·44, 56·97	15·0	14·09, 16·06	8·4	5·8, 11·4
Jordan, 2012						
0–1 years	69·4	66·55, 72·02	8·4	6·91, 10·22	3·4	2·2, 5·0
1–2 years	74·9	73·21, 76·56	4·8	4·00, 5·65	1·7	0·9, 2·5
2–5 years	80·7	79·09, 82·12	3·8	3·16, 4·64	2·4	1·4, 4·0
Total	76·3	75·27, 77·37	5·0	4·50, 5·58	2·4	1·5, 3·8
Mauritania, 2000–2001						
0–1 years	67·2	63·20, 70·91	5·4	3·48, 8·17	20·5	17·42, 24·01
1–2 years	74·0	70·12, 77·61	2·5	1·51, 4·13	14·2	11·53, 17·39
2–5 years	78·3	75·89, 80·46	2·6	1·86, 3·52	12·2	10·11, 14·70
Total	74·7	72·44, 76·74	3·2	2·48, 4·21	14·5	12·7, 15·9
Morocco, 2004						
0–1 years	44·3	41·10, 47·45	21·4	18·79, 24·34	12·3	10·37, 14·51
1–2 years	53·4	50·79, 56·00	14·7	13·07, 16·48	8·5	7·28, 9·99
2–5 years	64·7	62·37, 67·05	8·3	7·09, 9·60	10·5	9·03, 12·11
Total	56·4	54·80, 58·08	13·3	12·24, 14·35	10·0	8·8, 11·9
Tunisia, 1988						
0–1 years	68·4	64·05, 72·53	7·6	5·20, 10·86	6·8	4·87, 9·34
1–2 years	71·1	68·08, 73·94	6·3	4·92, 8·11	2·2	1·58, 3·02
2–4 years	64·0	60·3, 67·4	5·8	4·3, 7·8	3·8	2·1, 4·8
Total	70·3	67·38, 73·11	6·9	5·46, 8·69	3·1	2·45, 3·96
Turkey, 2013						
0–1 years	66·7	60·09, 70·84	9·9	5·96, 12·44	2·0	1·11, 4·83
1–2 years	64·3	62·80, 70·93	13·8	11·29, 16·66	1·4	0·8, 2·0
2–5 years	62·6	61·1, 66·4	10·9	8·51, 13·66	3·1	2·17, 4·78
Total	64·1	61·0, 66·2	11·8	9·9, 14·5	1·7	1·1, 2·5
Yemen, 2013						
0–1 years	66·9	64·91, 68·40	4·2	3·57, 5·07	22·9	21·36, 24·48
1–2 years	76·6	75·21, 77·95	1·5	1·21, 1·97	16·9	15·63, 18·15
2–5 years	81·8	80·29, 83·29	1·4	1·08, 1·88	13·1	11·74, 14·50
Total	76·7	75·60, 77·70	2·0	1·76, 2·38	16·6	15·66, 17·57

Survey-adjusted estimates based on Demographic and Health Surveys (1988–2016) data for 0–5 years except for Tunisia (0–4 years); sample sizes ranged from 1329 to 9478 based upon the country. Using the WHO child growth standards, wasting defined as weight-for-height age-z-score < –2; overweight defined as weight-for-height z-score ≥ 2; normal weight defined as weight-for-height z-score ≥ –2 and < 1.

While Yemen had a high prevalence of only wasting (16·6 % wasted and only 2·0 % overweight), other countries in the region had both types of unhealthy weight: In Egypt, Armenia and Morocco, the proportion of children who were *not* in the normal-weight category approached 50 %.

Sex patterns in the prevalence of unhealthy weight are shown in Table 2. Across countries, the prevalence of at least one measure of unhealthy weight was higher for boys than for girls. Morocco had the highest proportion of children either wasted or overweight for both boys (24·6 %) and girls (22·1 %); Jordan had the lowest proportion of unhealthy weight (boys: 8·0 %; girls 6·6 %). Sex differences in unhealthy weight were significant in Mauritania (boys: 19·9 %, girls: 15·5 %, *P* = 0·03) and Yemen (boys: 20·2 %, girls: 17·0 %, *P* = 0·04).

**Table 2:** Distribution across weight categories among boys and girls under age 5 years in the Middle East and North Africa region at the most recent year (%)

	Overweight		Wasting		Unhealthy weight		*P*
Armenia, 2015							
Boys	14·1	11·37, 17·27	3·2	2·19, 4·70	17·3	14·47, 20·49	0·59
Girls	12·5	9·93, 15·70	5·0	3·45, 7·16	17·5	14·51, 21·00	
Azerbaijan, 2006							
Boys	13·1	11·13, 15·25	6·7	5·30, 8·37	19·7	17·41, 22·27	0·05
Girls	10·9	9·03, 13·10	5·5	4·20, 7·19	16·4	14·14, 18·96	
Egypt, 2014							
Boys	15·5	14·32, 16·83	8·4	6·4, 7·9	23·9	22·45, 25·51	0·35
Girls	14·5	13·36, 15·77	8·5	5·4, 10·4	23·2	21·69, 24·70	
Jordan, 2012							
Boys	5·7	4·98, 6·58	2·2	1·79, 2·82	8·0	7·09, 8·96	0·42
Girls	4·2	3·58, 5·02	2·3	1·84, 2·92	6·6	5·73, 7·50	
Mauritania, 2000–2001							
Boys	3·2	2·33, 4·49	17·0	14·7, 19·53	19·9	18·07, 21·87	0·03
Girls	3·2	2·28, 4·56	12·3	10·43, 14·39	15·5	13·84, 17·37	
Morocco, 2004							
Boys	14·1	12·72, 15·68	10·5	9·24, 11·87	24·6	22·78, 26·55	0·06
Girls	12·4	11·08, 13·86	9·6	8·49, 10·95	22·1	20·31, 23·91	
Tunisia, 1988							
Boys	6·7	4·90, 8·97	3·4	2·54, 4·57	10·1	7·92, 12·70	0·67
Girls	7·2	5·48, 9·33	2·8	2·03, 3·86	10·0	8·13, 12·18	
Turkey, 2013							
Boys	13·0	12·3, 15·5	2·2	1·26, 3·43	15·3	12·8, 16·1	0·07
Girls	10·3	9·1, 12·1	2·1	1·11, 3·39	12·4	11·6, 15·7	
Yemen, 2013							
Boys	2·1	1·70, 2·54	18·2	16·91, 19·40	20·2	18·89, 21·58	0·04
Girls	2·0	1·64, 2·46	15·0	13·88, 16·23	17·0	15·84, 18·29	

Survey-adjusted estimates based on Demographic and Health Surveys (1988–2016) data for 0–5 years except for Tunisia (0–4 years); sample sizes ranged from 1329 to 9478 based upon the country. Using WHO child growth standards, wasting defined as weight-for-height age-z-score < –2; overweight defined as weight-for-height z-score ≥ 2; normal weight defined as weight-for-height z-score ≥ –2 and < 1. Unhealthy weight defined as wasted or overweight; *P*-values refer to the differences between boys and girls.

Table 3 illustrates trends over time in terms of the proportion of children in each weight category in each country at each timepoint of DHS data collection. Egypt, Morocco and Turkey experienced increases in the prevalence of overweight over time. The prevalence of wasting increased in three countries: Armenia, Egypt and Morocco. Yemen stayed constant in the prevalence of wasting over time and had a decrease in overweight. Egypt had increases in both wasting and overweight between 1988 and 2014 (2·1–8·4 % and 8·4–15·0 %, respectively), resulting in an increase in unhealthy weight from 10·6 % to 23·6 %. Jordan and Turkey are the only countries that experienced a substantial decrease in the prevalence of wasting, with 3·8 % (Turkey – 1993) and 4·0 % (Jordan – 1990) of children being wasted in the early 1990s and about half that number in the 2010s (1·7 % in Turkey – 2013 and 2·4 % in Jordan – 2013).

**Table 3:** Distribution across weight categories among children under age 5 years in the Middle East and North Africa region over time (%)

	Normal weight		Overweight		Wasting		Unhealthy weight	
Armenia								
2000	53·4	49·5, 55·3	15·8	13·77, 18·11	2·4	1·5, 3·6	18·2	15·91, 20·69
2005	66·2	64·46, 67·82	14·6	13·89, 15·49	5·0	3·9, 7·9	19·1	17·12, 20·20
2010	55·7	52·24, 59·18	15·3	13·26, 17·68	4·0	2·9, 6·6	19·4	17·08, 21·91
2015	57·7	54·29, 61·07	13·3	11·17, 15·85	4·1	3·02, 5·41	17·4	15·04, 20·01
Azerbaijan								
2006	64·2	62·05, 66·32	12·1	10·67, 13·57	6·1	5·14, 7·29	18·2	16·52, 19·96
Egypt								
1988	60·2	58·09, 62·22	8·4	7·34, 9·55	2·1	1·56, 2·87	10·6	9·30, 11·81
1992	58·4	56·48, 50·30	13·9	12·50, 15·37	3·0	1·8, 5·7	17·8	16·28, 19·34
1995	57·6	56·19, 58·27	13·6	12·63, 14·66	4·6	2·3, 6·2	19·4	18·33, 20·46
2000	52·7	49·89, 54·37	17·8	15·92, 19·89	2·5	1·6, 4·6	20·8	18·75, 23, 12
2003	73·6	69·45, 75·12	8·9	6·34, 10·89	4·0	2·2, 6·1	14·1	11·12, 16·98
2005	62·3	61·9, 64·5	13·3	12·0, 16·2	4·7	2·8, 5·4	18·1	16·7, 20·8
2008	46·3	44·91, 47·62	19·3	18·00, 20·57	7·4	6·60, 8·23	34·2	32·79, 35·60
2014	55·7	54·44, 56·97	15·0	14·09, 16·06	8·4	6·5, 10·3	23·6	22·35, 24·83
Jordan								
1990	65·8	64·32, 67·24	8·4	7·61, 9·26	4·0	3·39, 4·64	12·4	11·46, 13·33
1997	78·0	76·64, 79·21	4·3	3·75, 4·89	1·9	0·8, 3·1	6·6	5·92, 7·41
2002	77·3	76·08, 78·40	4·4	3·90, 5·04	2·0	1·4, 3·1	6·77	6·10, 7·49
2009	71·4	69·21, 73·49	6·6	5·28, 8·12	1·5	0·8, 2·3	8·1	6·73, 9·77
2012	76·3	75·27, 77·37	5·0	4·50, 5·58	2·4	1·0, 3·8	7·3	6·67, 7·96
Mauritania								
2000–2001	74·7	72·44, 76·74	3·2	2·48, 4·21	14·5	12·3, 16·8	17·9	16·11, 19·85
Morocco								
1987	71·8	70·44, 73·11	5·6	5·0, 6·4	3·9	2·6, 4·5	9·4	8·58, 10·31
1992	61·7	59·68, 63·62	10·7	9·64, 11·81	2·5	1·7, 3·9	13·2	12·04, 14·44
2004	56·4	54·80, 58·08	13·3	12·24, 14·35	10·0	8·2, 12·1	23·3	21·94, 24·76
Tunisia								
1988	70·3	67·38, 73·11	6·9	5·46, 8·69	3·1	2·45, 3·96	10·0	8·39, 11·92
Turkey								
1993	74·3	72·59, 75·98	4·8	4·10, 5·67	3·8	3·06, 4·75	8·6	7·65, 9·74
1998	74·1	72·00, 75·99	3·7	3·04, 4·61	3·0	2·37, 3·73	6·7	5·75, 7·84
2003	75·1	73·54, 76·63	9·8	8·72, 11·04	0·7	9·1, 11·2	11·9	9·75, 12·12
2013	64·1	61·0, 66·2	11·8	9·9, 14·5	1·7	1·2, 3·8	14·0	11·5, 15·6
Yemen								
1992	71·6	70, 86, 74·39	6·0	4·45, 7·23	13·1	12·22, 14·94	19·1	17·29, 20·40
2013	76·7	75·60, 77·70	2·0	1·76, 2·38	16·6	15·66, 17·57	18·6	17·65, 19·67

Survey-adjusted estimates based on Demographic and Health Surveys (1987–2016) data for 0–5 years except for Tunisia (0–4 years) and Egypt 1988 (0–4 years); sample sizes ranged from 1329 to 12 618 based upon the country. Using WHO child growth standards, wasting defined as weight-for-height age-z-score < –2; overweight defined as weight-for-height z-score ≥ 2; normal weight defined as weight-for-height z-score ≥ –2 and < 1; unhealthy weight defined as children who are wasted or overweight.

**Fig. 1:**
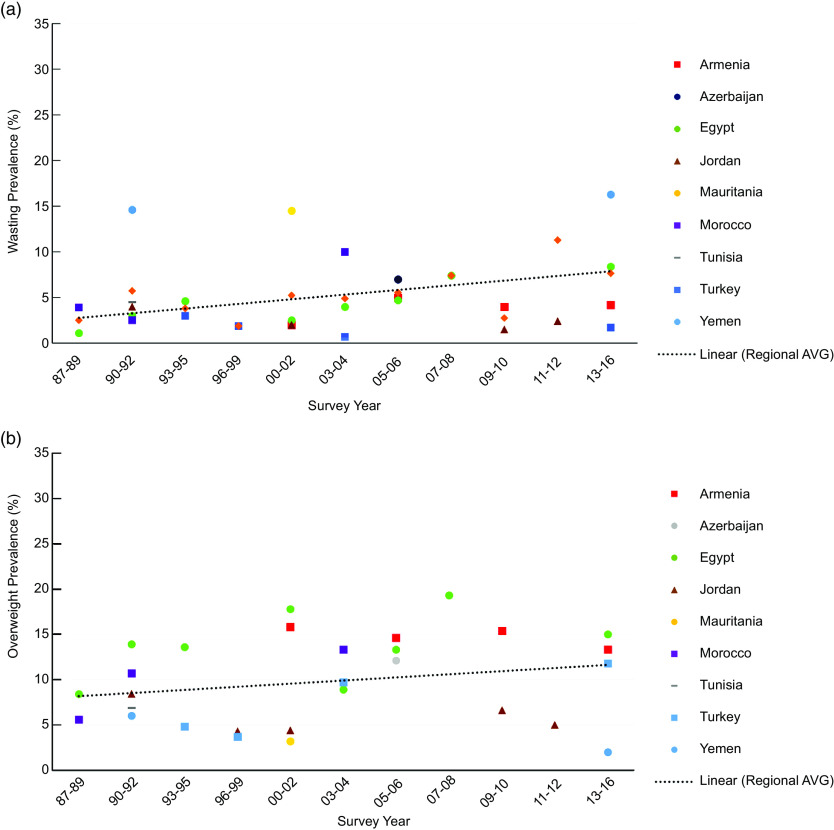
Prevalence of wasting (a) and overweight (b) among children under age 5 years in the Middle East and North African region over time Note: Survey-adjusted estimates based on Demographic and Health Survey (1987–2016) data for 0–5 years except for Tunisia (0–4 years) and Egypt 1988 (0–4 years); sample sizes ranged from 1329 to 12 618 based upon the country. Using WHO child growth standards, wasting defined as weight-for-height age-z-score < –2; overweight defined as weight-for-height z-score ≥ 2. Regional estimates based upon the average survey-adjusted prevalence for each range of years. *R* ^2^ = 0·489 (a), *R* ^2^ = 0·468 (b).

The overall trends in the prevalence of wasting (Fig. 1(a)) and overweight (Fig. 1(b)) in children under 5 in the region are shown in Fig. 1. The prevalence of both wasting and overweight increased in the region from 1988 to 2016 (*R* ^2^ = 0·489 and 0·468, respectively).

**Fig. 2:**
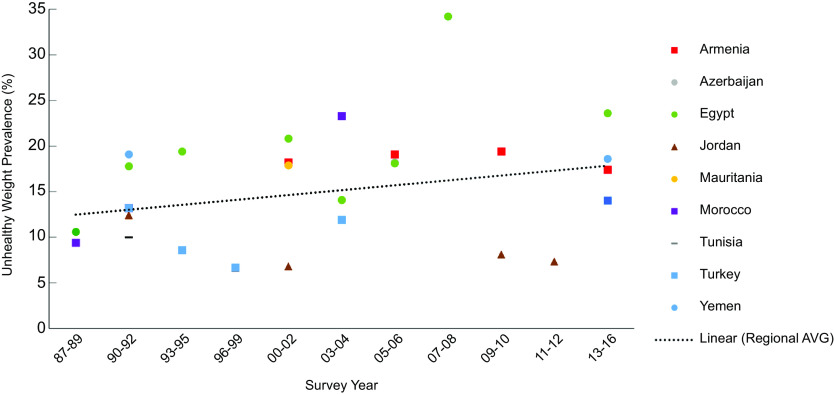
Prevalence of total unhealthy weight (wasting and overweight) among children under age 5 years in the Middle East and North African region over time Note: Survey-adjusted estimates based on Demographic and Health Survey (1987–2016) data for 0–5 years except for Tunisia (0–4 years) and Egypt 1988 (0–4 years); sample sizes ranged from 1329 to 12 618 based upon the country. Using WHO child growth standards, wasting defined as weight-for-height age-z-score < –2; overweight defined as weight-for-height z-score ≥ 2; unhealthy weight defined as children who are wasted or overweight. Regional estimates based upon the average survey-adjusted prevalence for each range of years. *R* ^2^ = 0·5979.

Figure 2 illustrates trends in the prevalence of unhealthy weight in children under 5 in the region with an overall regional trendline. The prevalence of unhealthy weight across the region increased from 10·0 % in 1988 to 18·4 % in 2016 (*R* ^2^ = 0·576).

**Fig. 3:**
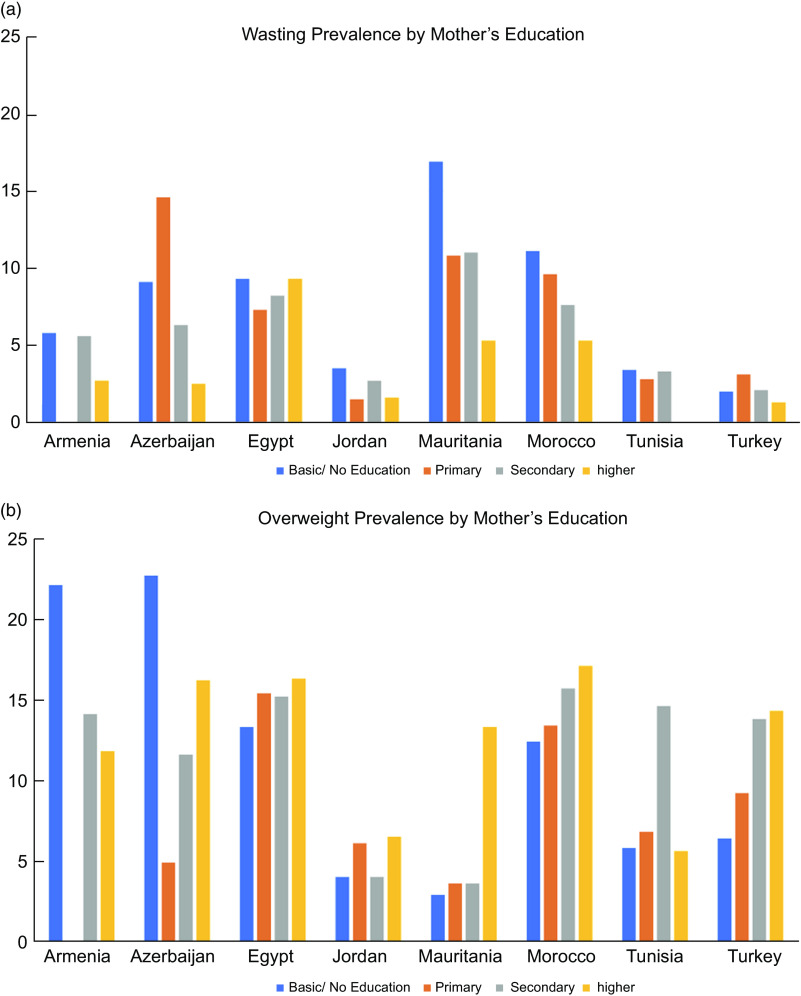
Prevalence of wasting (a) and overweight (b) among children under age 5 years in the Middle East and North African region stratified by mother’s education Note: Survey-adjusted estimates based on Demographic and Health Surveys (1988–2016) data for 0–5 years except for Tunisia (0–4 years) and Yemen due to missing mother’s education; sample sizes ranged from 1329 to 9478 based upon the country. Armenia’s primary and secondary categories were combined in mother’s education. Using the WHO child growth standards, wasting defined as weight-for-height age-z-score < –2; overweight defined as weight-for-height z-score ≥ 2.

Figure 3 illustrates social patterning of unhealthy weight in the region in terms of the prevalence of (Fig. 3(a)) wasting and (Fig. 3(b)) overweight in each country at the most recent time point stratified by mother’s education. The prevalence of wasting was highest among children born to mothers with basic or no education in Armenia, Jordan, Mauritania and Morocco and among children born to mothers with primary education in Azerbaijan and Turkey. Wasting was not patterned by mother’s education in Egypt and Tunisia.

Social patterns in overweight differ in countries across the region: the highest prevalence of overweight was among children born to mothers with basic or no education in Armenia and Azerbaijan but was highest among children born to mothers with higher education in Egypt, Mauritania, Morocco and Turkey and to mothers with secondary education in Tunisia. Overweight was not patterned by mother’s education in Jordan.

**Fig. 4:**
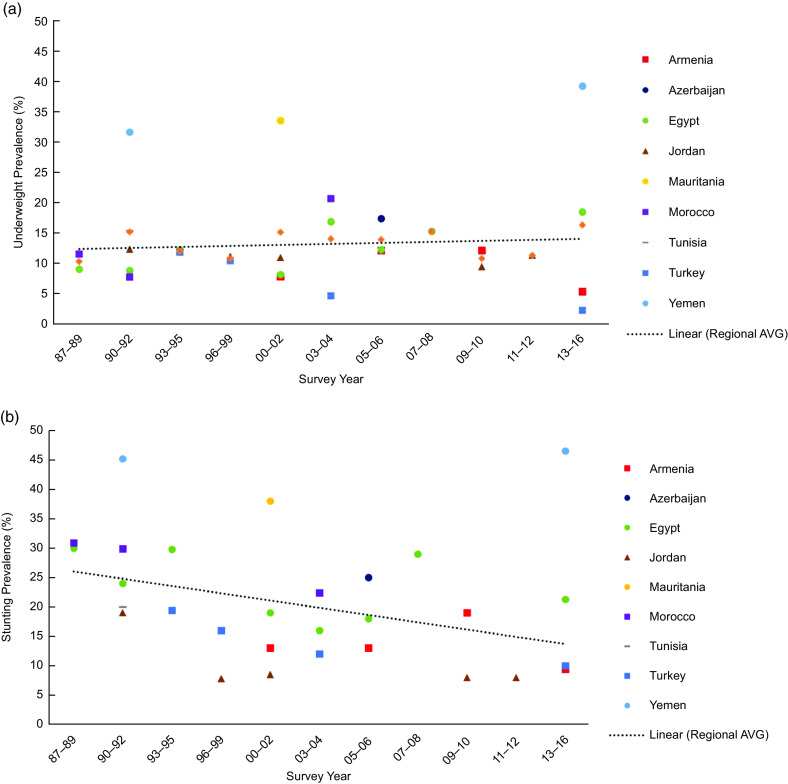
Prevalence of underweight (a) and stunting (b) among children under age 5 years in the Middle East and North African region over time Note: Survey-adjusted estimates based on Demographic and Health Survey (1987–2016) data for 0–5 years with the exception of Tunisia (0 to 4y) and Egypt 1988 (0–4 years); sample sizes ranged from 1329 to 12 618 based upon the country. Using World Health Organization child growth reference standards: underweight defined as BMI-z-score for age and sex < –2; stunting defined as height-z-score for age and sex < –2. Regional estimates based upon the average survey-adjusted prevalence for each range of years. *R* ^2^ = 0·436 (a), *R* ^2^ = 0·513 (b).

**Fig. 5:**
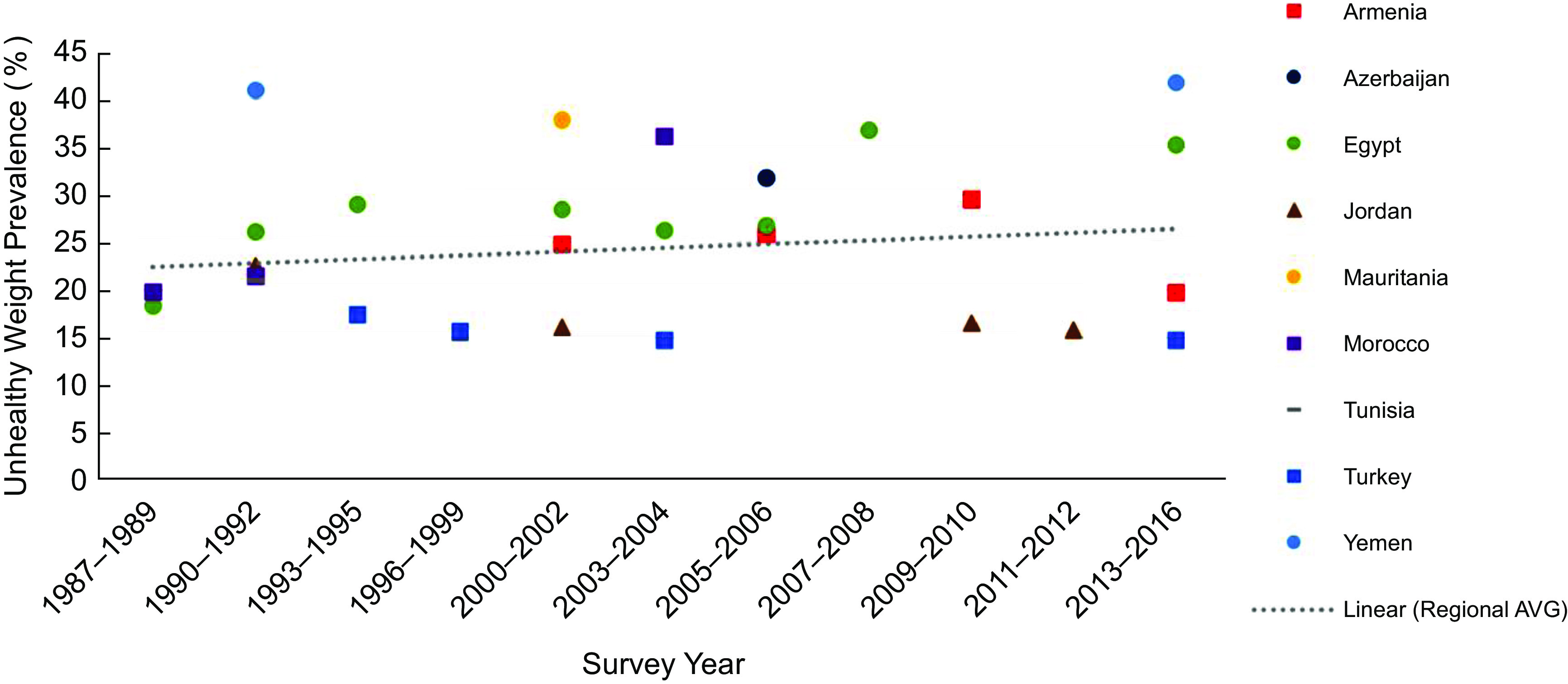
Prevalence of unhealthy weight (underweight and overweight) among children under age 5 years in the Middle East and North African region over time Note: Survey-adjusted estimates based on Demographic and Health Survey (1987–2016) data for 0–5 years with the exception of Tunisia (0–4 years) and Egypt 1988 (0–4 years); sample sizes ranged from 1329 to 12 618 based upon the country. Using World Health Organization child growth reference standards: underweight defined as BMI-z-score for age and sex < –2; overweight defined as BMI-*z*-score defined as ≥ 2; unhealthy weight defined as children who are underweight or overweight. Regional estimates based upon the average survey-adjusted prevalence for each range of years. *R* ^2^ = 0·5982.

In additional analyses, we also evaluated undernutrition in terms of underweight and stunting. The prevalence of underweight in children under age 5 years ranged between 39·2 % in Yemen (2013) and 2·2 % in Turkey (2013) (Table 4). The prevalence of underweight decreased with age, following patterns similar to that of wasting. Sex differences in underweight were significant in Tunisia (boys: 16·5 %, girls: 14·0 % χ^2^(3) = 10·8, *P* = 0·020) and Azerbaijan (boys: 18·6 %%, girls: 15·8 %, χ^2^(3) = 19·2, *P* = 0·002). The prevalence of stunting was lowest in Jordan (2012) at 8·0 % and highest in Yemen (2013) at 46·5 % (Table 5). Generally, the prevalence of stunting increased with age, except in Armenia and Jordan. The prevalence of stunting was higher for boys than girls in all nine countries, but sex differences were only significant in Jordan (boys: 9·0 %, girls: 6·0 %, *P* = 0·034). The prevalence of underweight increased over time similarly to the prevalence of wasting (Fig. 4(a)), while the prevalence of stunting in the region considerably declined over time (*R* ^2^ = 0·513) (Fig. 4(b)). Figure 5 illustrates trends in the prevalence of unhealthy weight, defined with an alternative measure, specifically underweight and overweight rather than wasting and overweigh. An overall regional trendline for this measure is also provided. Using this measure, the prevalence of unhealthy weight across the region has also increased from 22.1% in 1988 to 25.2 % in 2016 (*R* ^2^ = 0·589).

**Table 4:** Underweight among children under age 5 years in the Middle East and North African region at the most recent year of national data collection (%)

By age groups			By sex		
Armenia, 2015					
0–1 years	7·1	4·7, 10·6	Boys	4·0	2·9, 5·6
1–2 years	0·6	0·1, 2·5	Girls	6·8	5·2, 8·9
2–4 years	6·3	4·9, 8·0			
Total	5·3	4·3, 6·5			
Azerbaijan, 2006					
0–1 years	35·3	28·1, 37·2	Boys	18·6	15·6, 20·2
1–2 years	15·7	10·9, 17·8	Girls	15·8	13·5, 18·3
2–4 years	11·2	10·4, 14·2			
Total	17·3	15·2, 18·6			
Egypt, 2014					
0–1 years	24·9	19·2, 28·5	Boys	18·9	16·9, 20·1
1–2 years	18·9	14·9, 19·8	Girls	17·8	14·9, 20·1
2–4 years	15·9	11·1, 17·7			
Total	18·4	16·3, 19·1			
Jordan, 2012					
0–1 years	15·9	12·4, 20·1	Boys	11·3	9·6, 13·2
1–2 years	9·2	6·8, 12·4	Girls	11·4	9·6, 13·5
2–4 years	10·8	9·2, 12·6			
Total	11·3	10·0, 12·8			
Mauritania, 2000–2001					
0–1 years	46·0	42·0, 50·0	Boys	35·0	32·1, 37·9
1–2 years	30·5	27·0, 34·2	Girls	32·0	28·9, 35·2
2–4 years	29·1	26·0, 32·4			
Total	33·5	31·3, 35·8			
Morocco, 2004					
0–1 years	24·1	21·4, 27·0	Boys	20·7	18·8, 22·6
1–2 years	16·5	14·3, 18·9	Girls	20·5	18·8, 22·3
2–4 years	20·8	19·0, 22·7			
Total	20·6	19·2, 22·1			
Tunisia, 1988					
0–1 years	29·4	25·8, 33·4	Boys	16·5	14·3, 18·9
1–2 years	9·0	7·1, 11·3	Girls	14·0	12·0, 16·4
2–3 years	10·4	8·4, 12·9			
Total	15·3	13·7, 16·9			
Turkey, 2013					
0–1 years	2·0	1·5, 3·9	Boys	2·1	1·4, 3·0
1–2 years	1·1	0·8, 2·5	Girls	2·3	1·9, 3·3
2–4 years	2·6	1·4, 4·1			
Total	2·2	1·2, 3·8			
Yemen, 2013					
0–1 years	54·3	48·7, 57·8	Boys	38·5	35·9, 40·0
1–2 years	39·7	37·6, 42·7	Girls	39·0	37·0, 41·2
2–4 years	33·8	31·0, 36·5			
Total	39·2	37·5, 41·6			

Survey-adjusted estimates based on Demographic and Health Surveys (1988–2014) data for 0–5 years with the exception of Tunisia (0–4 years); sample sizes ranged from 1329 to 9478 based upon the country. Using the World Health Organization child growth reference standards: underweight defined as BMI z-score for age and sex < –2.

**Table 5:** Stunting among children under age 5 years in the Middle East and North African region at the most recent year of national data collection (%)

By age groups			By sex		
Armenia, 2015					
0–1 years	17·1	12·3, 19·1	Boys	10·9	6·7, 13·8
1–2 years	10·0	8·5, 11·9	Girls	7·8	6·2, 9·9
2–4 years	6·4	5·0, 8·2			
Total	9·4	7·3, 12·1			
Azerbaijan, 2006					
0–1 years	14·5	13·1, 15·2	Boys	26·7	23·6, 28·5
1–2 years	16·3	10·9, 17·7	Girls	23·4	20·5, 24·8
2–4 years	31·6	29·7, 34·1			
Total	25·0	22·5, 27·4			
Egypt, 2014					
0–1 years	18·9	15·2, 21·0	Boys	22·8	20·3, 24·9
1–2 years	21·3	18·7, 24·0	Girls	19·9	16·9, 21·8
2–4 years	22·5	19·6, 25·3			
Total	21·3	17·1, 23·4			
Jordan, 2012					
0–1 years	13·0	11·1, 15·4	Boys	9·0	7·3, 10·9
1–2 years	10·2	8·7, 13·1	Girls	6·0	4·9, 7·2
2–4 years	5·2	4·0, 7·1			
Total	8·0	6·2, 10·1			
Mauritania, 2000–2001					
0–1 years	15·4	13·1, 18·0	Boys	38·8	36·5, 41·1
1–2 years	41·5	37·9, 45·2	Girls	37·3	35·0, 39·7
2–4 years	47·0	44·7, 49·3			
Total	38·0	36·4, 39·7			
Morocco, 2004					
0–1 years	19·6	17·1, 22·0	Boys	23·9	22·3, 25·9
1–2 years	23·5	19·9, 25·9	Girls	21·0	19·4, 23·0
2–4 years	28·7	25·7, 31·1			
Total	22·4	20·2, 24·8			
Tunisia, 1988					
0–1 years	14·0	11·4, 17·1	Boys	20·7	17·9, 22·9
1–2 years	21·5	18·7, 24·7	Girls	19·4	16·4, 22·1
2–3 years	23·2	20·3, 26·5			
Total	20·0	18·3, 21·8			
Turkey, 2013					
0–1 years	5·6	3·9, 7·1	Boys	10·9	9·3, 15·5
1–2 years	9·9	7·1, 12·3	Girls	8·0	6·6, 11·3
2–4 years	12·2	11·1, 14·2			
Total	10·1	8·5, 12·2			
Yemen, 2013					
0–1 years	26·4	22·0, 29·9	Boys	47·6	44·1, 49·1
1–2 years	41·2	38·7, 44·3	Girls	45·4	42·2, 47·9
2–4 years	54·0	51·2, 56·8			
Total	46·5	42·3, 50·1			

Survey-adjusted estimates based on Demographic and Health Surveys (1988–2014) data for 0–5 years with the exception of Tunisia (0–4 years); sample sizes ranged from 1329 to 9478 based upon the country. Using the World Health Organization child growth reference standards: stunting defined as height-for-age-z-score < –2.

## Discussion

Among children under the age of 5 years in the MENA region, wasting and overweight represent the double burden of unhealthy weight. Both under- and overnutrition at young ages are of concern, as nutrition and growth patterns during the first years of life may have long-term health implications^(37)^. The prevalence of unhealthy weight among children under the age of 5 years varied across the MENA region at the most recent time point from 7·3 % in Jordan to 18·6 % in Yemen.

Overweight is emerging even during the first 5 years of life in this region. Overweight is accompanied by the continuing presence of wasting in the region, which is highest in Yemen and Mauritania. This phenomenon, in conjunction with rising obesity over time, particularly among women in Egypt, Jordan, Morocco and Turkey, is cause for concern^(38)^. Previously, we showed household-level concordance of malnutrition among mothers and children in MENA countries also using DHS data: 8·6 % of households experienced double burden (one underweight and one overweight among mother and child), 11 % experienced overnutrition (both overweight) and 0·2 % experienced undernutrition (both underweight)^(39)^. In the present paper, we add more evidence of increasing overweight among, even among very young children. Even with these increases in overweight, the prevalence of wasting in the region has also increased over time.

Across countries, boys experienced unhealthy weight more often than girls. Overweight was higher in boys than in girls in five out of the nine countries. Higher overnutrition among boys has been observed among older children in other settings, for example, in Pakistan^(40)^ and Kenya^(41)^. In all countries in this study but Armenia and Jordan, a higher proportion of boys than girls were also underweight, a pattern also reported for this age group across Africa^(42)^. The slightly higher risk of underweight among boys may be linked with boys’ higher susceptibility to infectious diseases^(40,42)^. If the high demand for nutrients due to boys’ growth trajectories is not met adequately, they can experience more deficits and at younger ages than girls, explaining the link between a child’s sex and wasting^(43,44)^.

This study includes a number of limitations. An underlying challenge to our and others’ work is the lack of health surveillance data in the region. The region consists of high-, middle- and low-income countries, but only middle- and low-income countries participate in the DHS, so we did not document the full breadth of the region, excluding high-income countries. This exclusion likely entails that countries with low prevalence of underweight and high prevalence of overweight among young children are excluded; indeed, the countries with the highest obesity prevalence among adults are excluded: Libya, Qatar and Kuwait^(8)^. Even among countries with DHS data, a few countries had data that are already two decades old, providing an outdated picture of population health. Globally, the nutrition transition happens in a nonlinear manner even within countries due to socio-economic, environmental and cultural diversity. We examined social distribution of unhealthy weight by mother’s education, but other social contexts may also be important. We note a substantial decrease in overweight prevalence in Egypt from 2000 to 2003 (17·8–8·9 %) and then back to 13·3 % in 2005. These changes may result from differences in sampling and data collection methods; indeed, 2000 and 2005 were full DHS surveys, whereas 2003 was an Interim DHS subsample.

Research on non-communicable diseases has become a priority only recently in many countries of the MENA region^(22,23)^. It will be important to have strong surveillance efforts across the region, providing timely, regular and quality data. Indeed, more data are forthcoming in the region. For example, Qatar’s Nation Health Strategy (2018–2022) is aimed at obesity prevention and is developing projects such as the biobank and Active Healthy Kids Report Card^(45,46)^; Kuwait has programmes focused on overnutrition among children and adolescents^(47)^.

Several of the results presented here relate to historical contexts. For example, the high levels of wasting in Yemen are likely due to the fact that the country has struggled with famine for close to a decade. Turkey made a commitment to improving undernutrition among children in the 1990s, which is demonstrated to have been effective by decreases in underweight over time^(48)^.

Using over 20 years of nationally representative survey, this study characterises unhealthy weight on both ends of the spectrum – wasting and overweight – in the MENA region. Both under- and overnutrition result from dysregulation of energy balance, and thus, prevention efforts should aim to consider both^(49,50)^. Some policies in the region appear discordant with social, economic and political circumstances, for example, food fortification of rice flour in Egypt^(23,24)^. More recent policies in the area have focused on both sides of the malnutrition burden^(24,25)^. A recent report of the nutrition landscape in Egypt is a great example of giving a thorough review of past and current interventions and programme^(24)^. The report includes an analysis of interventions that could identify windows of vulnerability to both overweight and underweight, providing an example of a policy programme for both types of unhealthy weight.

This study demonstrates the need to evaluate and develop programmes to address both wasting and overweight among children. These results show increases in overweight and wasting in children under 5 years across the region overall, with considerable variability by country across and among socio-economic groups within countries. Countries in the region must advance programmes that reduce undernutrition while not overlooking or inadvertently promoting overnutrition.
